# COVID-19 Psychiatric Inpatient Unit- experience and challenges

**DOI:** 10.1192/j.eurpsy.2022.1326

**Published:** 2022-09-01

**Authors:** B. Mesquita, A. Fraga, M. Albuquerque, P. Espada-Santos, J. Facucho-Oliveira, S. Paulino, M. Costa

**Affiliations:** Hospital de Cascais, Psychiatry, Alcabideche, Portugal

**Keywords:** Covid-19, bipolar disorder, Psychiatric Inpatient Unit, Psychotic disorder

## Abstract

**Introduction:**

On January 2021 the Department of Psychiatry became the only unit exclusively dedicated to COVID patients with severe mental illness in acute decompensation. Only patients in risk of rapid medical deterioration were excluded and forwarded to intensive care.

**Objectives:**

Discussion of this unprecedented experience.

**Methods:**

Analysis of 28 patients hospitalized during 3 months with both an acute psychiatric disorder and an SARS-CoV-2 infection; description of the multidisciplinary intervention made.

**Results:**

Our sample was characterized by a majority of patients with an acute psychotic episode derived from a schizophrenia spectrum disorder (42%) or a bipolar affective disorder (21%). Only 3% of the patients had a diagnosis of severe major depressive disorder. And 10% of patients developed severe respiratory symptoms requiring oxygen or urgent transfer to COVID medical wards. Most patients presented periods of psychomotor agitation, lack of impulse control and self-aggression. Psychopharmacological and psychotherapeutic interventions had to be adapted to these unusual conditions. Most of them had already gone through a period of isolation in the buffer ward created to exclude false negatives, which promoted atypical deliriums and symptoms of post-traumatic stress. The psychiatric team was faced with the emergent need to adapt an intervention model based on trust to a model that had to prioritize physical safety.

**Conclusions:**

The pandemic experience was transformative for all who lived through it. From the challenge perspective, it may have been enriching. But the maintained confrontation with the antithesis of therapy, defined by “caring, supporting, communicating, approaching”, was devastating in ways that we consider essential to be debated.

**Disclosure:**

No significant relationships.

